# Hepatic Oxidative Stress, Genotoxicity and Vascular Dysfunction in Lean or Obese Zucker Rats

**DOI:** 10.1371/journal.pone.0118773

**Published:** 2015-03-04

**Authors:** Mille Løhr, Janne K. Folkmann, Majid Sheykhzade, Lars J. Jensen, Ali Kermanizadeh, Steffen Loft, Peter Møller

**Affiliations:** 1 Section of Environmental Health, Department of Public Health, University of Copenhagen, Øster Farimagsgade 5A, DK-1014 Copenhagen K, Denmark; 2 Department of Drug Design and Pharmacology, Faculty of Health and Medical Sciences, University of Copenhagen, Universitetsparken 2, DK-2100 Copenhagen Ø, Denmark; 3 Department of Veterinary Clinical and Animal Sciences, Faculty of Health and Medical Sciences, Grønnegårdsvej 7, 1870 Frederiksberg C, Denmark

## Abstract

Metabolic syndrome is associated with increased risk of cardiovascular disease, which could be related to oxidative stress. Here, we investigated the associations between hepatic oxidative stress and vascular function in pressurized mesenteric arteries from lean and obese Zucker rats at 14, 24 and 37 weeks of age. Obese Zucker rats had more hepatic fat accumulation than their lean counterparts. Nevertheless, the obese rats had unaltered age-related level of hepatic oxidatively damaged DNA in terms of formamidopyrimidine DNA glycosylase (FPG) or human oxoguanine DNA glycosylase (hOGG1) sensitive sites as measured by the comet assay. There were decreasing levels of oxidatively damaged DNA with age in the liver of lean rats, which occurred concurrently with increased expression of Ogg1. The 37 week old lean rats also had higher expression level of Hmox1 and elevated levels of DNA strand breaks in the liver. Still, both strain of rats had increased protein level of HMOX-1 in the liver at 37 weeks. The external and lumen diameters of mesenteric arteries increased with age in obese Zucker rats with no change in media cross-sectional area, indicating outward re-modelling without hypertrophy of the vascular wall. There was increased maximal response to acetylcholine-mediated endothelium-dependent vasodilatation in both strains of rats. Collectively, the results indicate that obese Zucker rats only displayed a modest mesenteric vascular dysfunction, with no increase in hepatic oxidative stress-generated DNA damage despite substantial hepatic steatosis.

## Introduction

Metabolic syndrome is a common disorder with a number of risk factors, including obesity, insulin resistance, high plasma concentration of lipids, fasting hyperglycaemia and hypertension [1]. Moreover, metabolic syndrome is intricately associated with non-alcoholic fatty liver disease [2]. Although this predisposition is considered to be a relatively benign condition, and may lead to non-alcoholic steatohepatitis entailing both inflammation and oxidative stress. There are associations between elevated free fatty acids levels in serum, insulin resistance and endothelial dysfunction where oxidative stress also plays a role [3]. Endothelial dysfunction can be measured in various segments of the arterial side of the vascular system. With regards to the associated toxic effects in the liver and high energy intake, the vasomotor dysfunction of mesenteric arteries is particularly interesting considering the splanchnic hemodynamics and portal blood flow. Indeed, hepatic steatosis has been associated with increased portal pressure and intrahepatic endothelial dysfunction in a rat model [4]. Moreover, liver cirrhosis is often associated with endothelial dysfunction in the mesenteric arteries in humans [5].

We hypothesized that hepatic oxidative stress and steatosis related to hyperphagia would be associated with vascular dysfunction due to increased portal pressure and with reverse failure to the mesenteric vascular bed. This can display as altered vasomotor function or remodelling of mesenteric arteries. Outward remodelling (i.e. increased vessel size) is described in the context of atherosclerosis where a compensatory enlargement of vessel counteracts protrusion of plaques into the vessel lumen. However, outward remodelling may also occur due to increased blood flow in non-atherosclerotic vessels [6]. This can be investigated in obese Zucker rats, which develop characteristics similar to metabolic syndrome in humans. These rats suffer from hyperphagia due to a lack of functional leptin receptors leading to obesity, hyperlipidaemia, mild glucose intolerance and hyperinsulinaemia, as well as hypertension and non-alcoholic fatty liver disease [7]. Oxidative stress and inflammation are often implicated in the hepatic response in Zucker rats [8]. Here, we measured signs of oxidative stress by the expression level of *heme oxygenase (decycling) 1* (*Hmox1*) that contains an anti-oxidant response element in the promoter region and it is up-regulated in response to oxidative stress [9]. This was accompanied by measurement of HMOX-1 protein in the Zucker rat livers. In addition, the levels of oxidatively damaged DNA in the liver as strand breaks (SB, including alkali-labile sites) and formamidopyrimidine DNA glycosylase (FPG) and human oxoguanine DNA glycosylase (hOGG1) sensitive sites was quantified. The FPG enzyme detects 8-oxo-7,8-dihydroguanine and ring-opened formamidopyrimidine lesions, whereas the hOGG1 enzyme only detects the former. 8-Oxo-7,8-dihydroguanine is a pre-mutagenic lesion in DNA and it has been observed in cohort studies that increased excretion of this nucelobase or 8-oxo-7,8-dihydro-2’-deoxyguanosine (8-oxodG) in urine which is associated with risk of lung or breast cancer in humans [10–12]. We also measured the expression level of *N-methylpurine DNA glycosylase* (*Mpg*) that is involved in the repair of lipid peroxidation-derived exocyclic DNA adducts [13], as well as assessing the expression of *sterol regulating element-binding protein 2* (*Srebp-2*) involved in the metabolism of cholesterol in the liver [14]. In addition, alterations in the transport capacity of sterols were assessed by expression of ATP-binding cassette, sub-family G (WHITE), member 5 (*Abcg5*) and 8 (*Abcg8*), which encode transporters of cholesterol excretion into bile [15,16]. The specific purpose of the study was to investigate the effects of age on hepatic oxidative stress and vasomotor function and vessel wall remodelling in isolated mesenteric resistance arteries of lean and obese Zucker rats.

## Materials and Methods

### Housing

Age-matched lean and obese Zucker female rats (Charles River, Germany) were acclimatized for at least one week before starting the experimental protocol. They were housed in an animal facility with 12/12 h light/dark cycles, optimal temperature (22–24°C) and humidity (40–70%). All rats had free access to tap water and Standard Altromin no. 1314 rat chow (Altromin, Lage, Germany). All animal procedures followed the guidelines for the care and handling of laboratory animals and were ethically approved (The Danish government, and the Animal Experiment Inspectorate, under the Ministry of justice (no.2006/561–1161)). The rats were euthanized at 14, 24 or 37 weeks of age. The rats from this study have previously served as a control group in experiments on oral exposure to carbon black [17,18]. There were 8 rats in each group of lean and obese Zucker rats. However, for a few rats the mesenteric arteries could not be used due to the inclusion or exclusion criteria and the reported number of animal in some groups may be smaller for the mesenteric vasomotor response. The rats were housed in metabolic cages for 24 h prior to euthanasia. They were anaesthetized with Hypnorm/Dormicum (0.3 ml/100 g body weight) and blood samples taken from the tip of the tail. The rats were killed by cervical dislocation and mesenterium removed and processed on the same day, whereas the liver was snap frozen and stored at -80°C for later analysis of DNA damage, gene expression and Western blot.

### Measurement of DNA damage by the comet assay

The level of SB, FPG- and hOGG1-sensitive sites was measured by the comet assay as previously described [19]. The cells were embedded into 0.75% agarose on gel bonds and lyzed either for 1 h or overnight in lysis solution (2.5 M NaCl, 100 mM Na_2_EDTA, 10 mM Trizma base, pH 10.0). The gel bonds were washed 3×5 min in a buffer (40 mM HEPES, 0.1 M KCl, 0.5 mM Na_2_EDTA, 200 μg/ml BSA, pH 8.0). FPG or hOGG1 enzyme was added to the relevant gel bonds and incubated for 45 min at 37°C. The FPG enzyme was a gift from Professor Andrew Collins (University of Oslo, Norway), and the hOGG1 enzyme was obtained from New England Biolabs (Ipswich, MA, USA). The gel bonds were then transferred to alkaline solution for 40 min (1 mM Na_2_EDTA, 300 mM NaOH, pH > 13.0) and were subsequently subjected to electrophoresis for 20 min in the same buffer at 300 mA and 0.83 V/cm (from anode to cathode). After electrophoresis, gel bonds were washed 3×5 min in neutralization buffer (0.4 M Trizma base, pH 7.5), rinsed with water and immersed in 96% ethanol overnight. Gel bonds were air-dried, stained with YOYO-1 (Molecular probes, Eugene, OR, USA) and visually scored in a blinded fashion. The levels of DNA damage were obtained by scoring 100 nuclei/gel in 2 gels. We used a five-class scoring system and transformed to lesions/10^6^ bp with a calibration curve where one arbitrary unit corresponds to 0.0273 lesions/10^6^ bp [20]. The level of FPG or hOGG1 sensitive sites was calculated as the difference in DNA damage between slides that had been treated with the FPG or hOGG1 enzyme and buffer. We used Ro19-8022 and light exposed monocytic THP-1 cells as reference controls, which has been used as control in comet assay validation trials [21–23]. Ro19-8022 was a gift from F. Hoffmann-La Roche (Basel, Switzerland).The levels of SB, FPG- and hOGG1-sensitive sites were 0.51 ± 0.31, 0.77 ± 0.19 and 0.48 ± 0.12 lesions/10^6^ base pairs, respectively.

### Measurement of gene expression

Gene expression in rat livers was measured by quantative real-time-PCR with 18S as reference gene as described previously [19]. The Gene IDs are as follows: *Abcg5* (Gene ID: 114628), *Abcg8* (Gene ID: 155192), *Hmox1* (Gene ID: 24451), *Mpg* (Gene ID: 24561), *Ogg1* (Gene ID: 81528) and *Srebp-2* (Gene ID: 50671). The quantification of gene expression was determined by real-time PCR using the Taqman gene expression assay with deoxyribonuclease (DNase) treatment to degrade genomic DNA. The probe and primers were: *Abcg5* (Rn00587092_m1), *Abcg8* (Rn00590367_m1), *Hmox1* (Rn00561387_m1), *Mpg* (Rn00561506_m1), *Ogg1* (Rn00578409_m1) and *Srebp2* (Rn01502638_m1) and eukaryotic 18S rRNA (X03205.1) (Applied Biosystems, Life Technologies Europe BV, Nærum, Denmark). The level of mRNA expression normalized to the level of 18S rRNA was calculated as 2^-ΔCt^.

### Determination of HMOX1 protein levels in liver lysates

For western blot analysis five animals were chosen from each group in random and total protein extracted from the livers and concentrations measured by Coomassie Plus Bradford assay reagent (Thermo Scientific, USA). β-mercaptoethanol was added to a final concentration of 5%, after which each sample was denatured by heating for 10 min (95°C). Next, 40 μg of protein from each sample was added to a 10% sodium dodecyl sulphate (SDS)-polyacrylamide gel electrophoresis (PAGE) gel. Electrophoresis productions were transferred onto polyvinylidene difluoride (PVDF) membranes (Bio-rad, USA), blocked with 5% bovine serum albumin/0.1% Tween-20, incubated with primary antibody (1:1000) (Sigma, UK) and HRP-conjugated secondary antibody (1:5000) (AbD Serotec, UK), respectively. The proteins were visualized using the ECL Western Blotting substrate kit (Abcam, USA) according to its manufacturer’s instructions. Finally, immunoblotting signals were quantitated using Image Studio 4.0 (LI-COR Biotechnology, USA).

### Vasomotor function

The mesenterium was pinned to the bottom of a dish, and a 5–8 mm long segment of the second-order branch of the mesenteric artery was dissected free from fat and surrounding tissue. Arterial segments were transferred to a chamber of a pressure myograph (Pressure myograph 110P, Danish Myo Technology, Århus, Denmark), containing bicarbonate-buffered physiological salt solution (PSS: 119 mM NaCl, 25 mM NaHCO_3_, 4.7 mM KCL, 1.18 mM KH_2_PO_4_, 1.17 mM MgSO_4_ · 7 H_2_O, 1.5 mM CaCl_2_ · 2H_2_O, 0.027 mM ethylene diamine tetraacetic acid and 5.5 mM glucose), which was kept at a constant temperature of 37°C and continuously aerated with a gas mixture of 5% CO_2_ and 95% O_2_ to maintain a pH of 7.4. At one end, the vessel was cannulated with the inflow pipette and tied with 11–0 nylon surgical thread. To flush blood out of the vessel lumen the inflow pressure was gently raised to 20 mmHg. At the other end, the vessel was cannulated with the outflow pipette and tied with 11–0 nylon surgical thread. After mounting, the chamber was transferred to the stage of an inverted microscope and the pressure was gently raised to 20 mmHg in PSS heated to 37°C and oxygenated with a gas mixture of 5% CO_2_ and 95% O_2_. After equilibration for 15 min the pressure was gently raised to 180 mmHg (10-mmHg increase in each step), at which point the vessel length was adjusted to avoid any buckling of the vessel. Thereafter the vessel was equilibrated at 80 mm Hg to generate spontaneous myogenic tone. The vessel segment was viewed through an inverted microscope and the vessel diameter evaluated continuously via a video microscope and analysed with MyoView1.2P (Danish Myo Technology, Århus, Denmark).

All experiments were performed under no-flow conditions where inlet and outlet pressures were equal. After equilibration at 80 mmHg for 20 min the pressure was set to 60 mmHg. The vessel segment was contracted (depolarised) with KPSS (125 mM K^+^) (same composition as PSS with the exception of the NaCl substituted with KCl on an equimolar basis) on three occasions separated by PSS washouts. This was done to verify functionality of the vessel segments, and the reproducibility of evoked contractions, and to deplete the sympathetic nerve endings of neurotransmitters (in particular noradrenaline). The endothelial function of the vessels was determined by pre-contracting the vessel segment with 2 × 10^-5^ M prostaglandin 2α and then performing cumulative concentration-response curve with acetylcholine (10^-10^–10^-4^ M with log-unit increments) on a stable pre-contraction tone induced by prostaglandin 2α. The presence of functional endothelium was assessed by the ability of acetylcholine to induce an increase in maximal relaxation by 40% in the pre-contracted vessels.

All steps in the concentration-response curve were collected in a graph with time as function of vessel diameter with a steady state value determined for each step. All the calculated values of vessel diameter were subtracted from the passive diameter (at 60 mmHg) and vasodilatation was expressed as the percentage of the pre-contracted diameter. The EC_50_ and E_max_ values were calculated using GraphPad Prism version 5.02 (San Diego, CA, USA). The data were fitted to sigmoid curves with variable slopes using non-linear regression analysis.

In order to determine the active pressure-diameter relationship the vessel segment was washed three times with PSS and the pressure was raised from 20 mmHg to 180 mmHg in 20-mmHg steps. Finally, the pressure steps were repeated in the presence of Ca^2+^-free PSS to construct a passive pressure curve. The pressure-diameter relationship was constructed from all increments in the pressure curve. Each pressure step was collected in a graph with time as a function of vessel diameter and a steady value was determined for each step.

### Statistical analysis

The results were analysed by two-way ANOVA with age and strain as categorical variables. Variance of homogeneity was assessed by Levene’s test. Results on HMOX-1 protein expression lever in liver tissue was analysed by two-way non-parametric ANOVA. For the pressure curves area in the assessment of vasodilatation, the area under the curve (AUC) were calculated and used for statistical analysis. All data are presented as mean ± SEM. The dose-response curves were analysed by ANOVA test.

## Results

### Body weight, hepatic lipid load, serum concentrations of non-fasting insulin, cholesterol and triglycerides are increased in obese Zucker rats

Table 1 displays a summary of body weight, hepatic lipid load and serum concentrations of non-fasting glucose, insulin, cholesterol, and alanine aminotransferase (ALT) (reproduced from previous publications) [17,18]. Both the lean and obese Zucker rats increased their body weight, although the obese Zucker rats had substantially higher body weight than the lean counterparts. The concentration of triglycerides and cholesterol were significantly higher in the obese Zucker rats at all ages. On the other hand, there was no difference in the serum concentration of non-fasting glucose between the obese and lean Zucker rats. All urine samples tested negative for glucose. The serum insulin concentrations declined with age in the obese Zucker rats.

**Table 1:** Body weight, hepatic lipid load and serum concentrations of triglycerides, cholesterol, non-fasting glucose, insulin and ALT in lean and obese Zucker rats.

End point	Age group	Lean rats	Obese rats
Body weight (g)	14 wk	220.4±4.4	416.9±12.6*
	24 wk	256.3±6.5	579.3±19.6*
	37 wk	281.9±7.3	695.0±29.7*
Triglyceride (mM)	14 wk	1.09±0.17	12.32±3.55*
	24 wk	1.08±0.16	18.73±4.84*
	37 wk	1.69±0.33	20.51±5.05*
Cholesterol (mM)	14 wk	2.18±0.073	3.25±0.23*
	24 wk	2.11±0.16	4.69±1.23*
	37 wk	2.49±0.17	7.67±1.46*
Glucose (mM)	14 wk	8.09±0.35	7.33±0.54
	24 wk	7.28±0.23	8.40±0.52
	37 wk	7.10±0.20	7.79±0.44
Insulin (μg/L)	14 wk	0.85±0.10	6.23±1.14*
	24 wk	0.65±0.07	1.99±0.26*
	37 wk	1.04±1.01	1.31±0.20
ALT (U/ml)	14 wk	91.4 ± 14.4	53.9 ± 4.2
	24 wk	54.4 ± 7.1	97.0 ± 25.7
	37 wk	85.6 ± 14.3	57.9 ±11.9
Lipid accumulation (fold)	14 wk	1.0±0.2	3.1 ± 0.1*
	24 wk	2.6 ± 0.2	3.6 ± 0.2*
	37 wk	2.8 ± 0.2	3.4 ± 0.1*

The results have been reported previously [17,18]. Lipid accumulation was assessed by Oil Red staining of liver tissue sections.

*P<0.05 compared to lean rats at the same age. The results is presented as the mean ± SEM (n = 6–8).

Both strains of rats had age-dependent increased lipid load in the liver, with these accumulations most evident in the obese rats at all ages. Nevertheless, there were unaltered serum levels of ALT in both the lean and obese Zucker rats.

### Lean Zucker rats have an age-associated increase in levels of DNA strand breaks and decreased levels of FPG-sensitive sites in the liver

**Fig 1:**
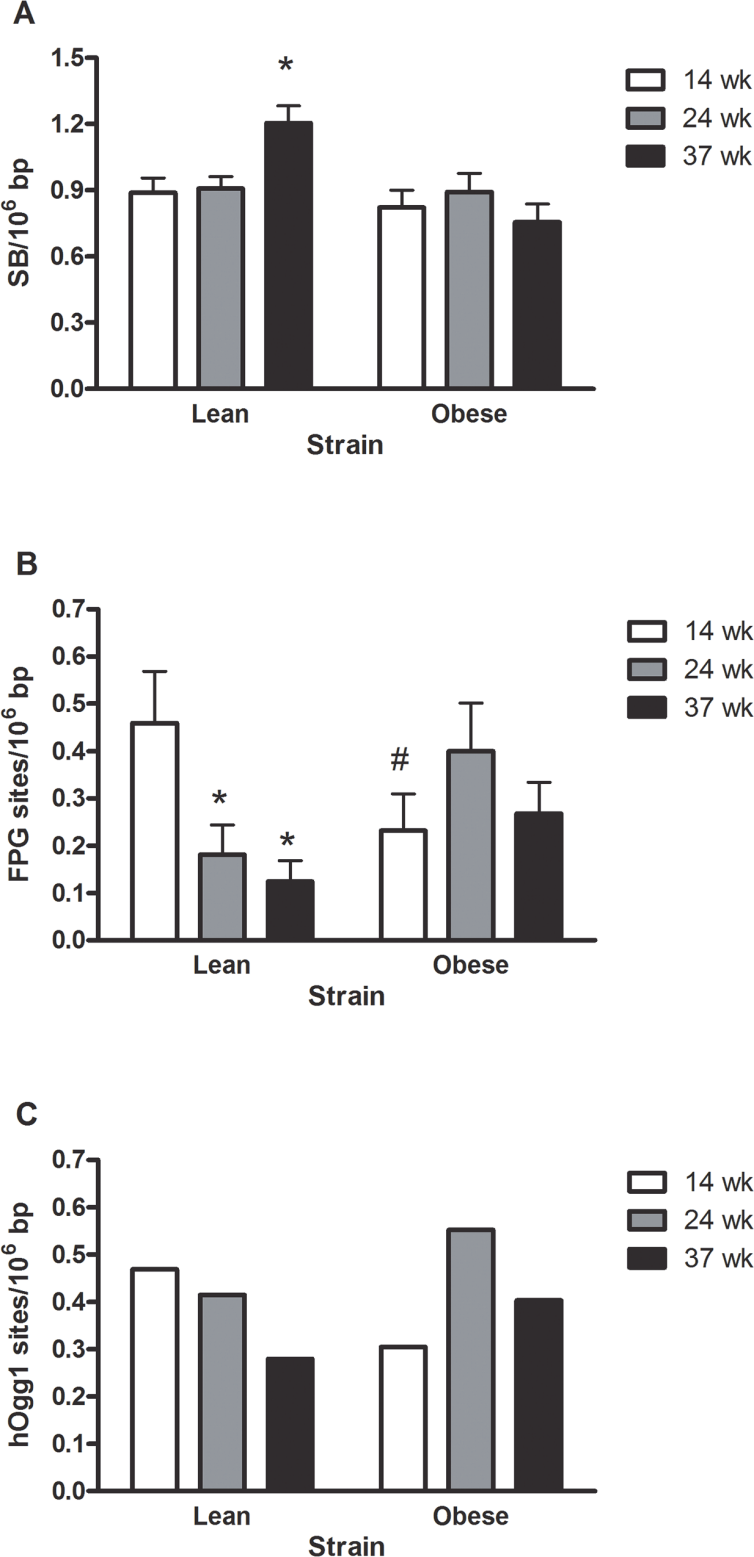
DNA strand breaks (A), FPG- (B) and hOGG1-sensitive sites (C) in the liver of lean or obese Zucker rats. The results are presented as mean and SEM (n = 7–8 per group). *P<0.05 compared to same strain of rat. ^#^P<0.05 compared to the lean counterpart at the same age.

There was increased level of DNA strand breaks in the lean rats at 37 weeks as compared to 14 weeks old lean rats and the 37 weeks old obese rats (Fig. 1A, P<0.05 for interaction between age and strain). The level of FPG-sensitive sites decreased with increasing age in the lean rats. The obese rats at 14 weeks had lower levels of FPG-sensitive sites than the lean rats at 14 weeks of age (Fig. 1B, P<0.05 for interaction between age and strain). There was no statistically significant changes in the levels of hOGG1-sensitive sites (Fig. 1C, P<0.10).

### Lean rats have increased gene expression of *Hmox1* and *Ogg1* in the liver

**Fig 2:**
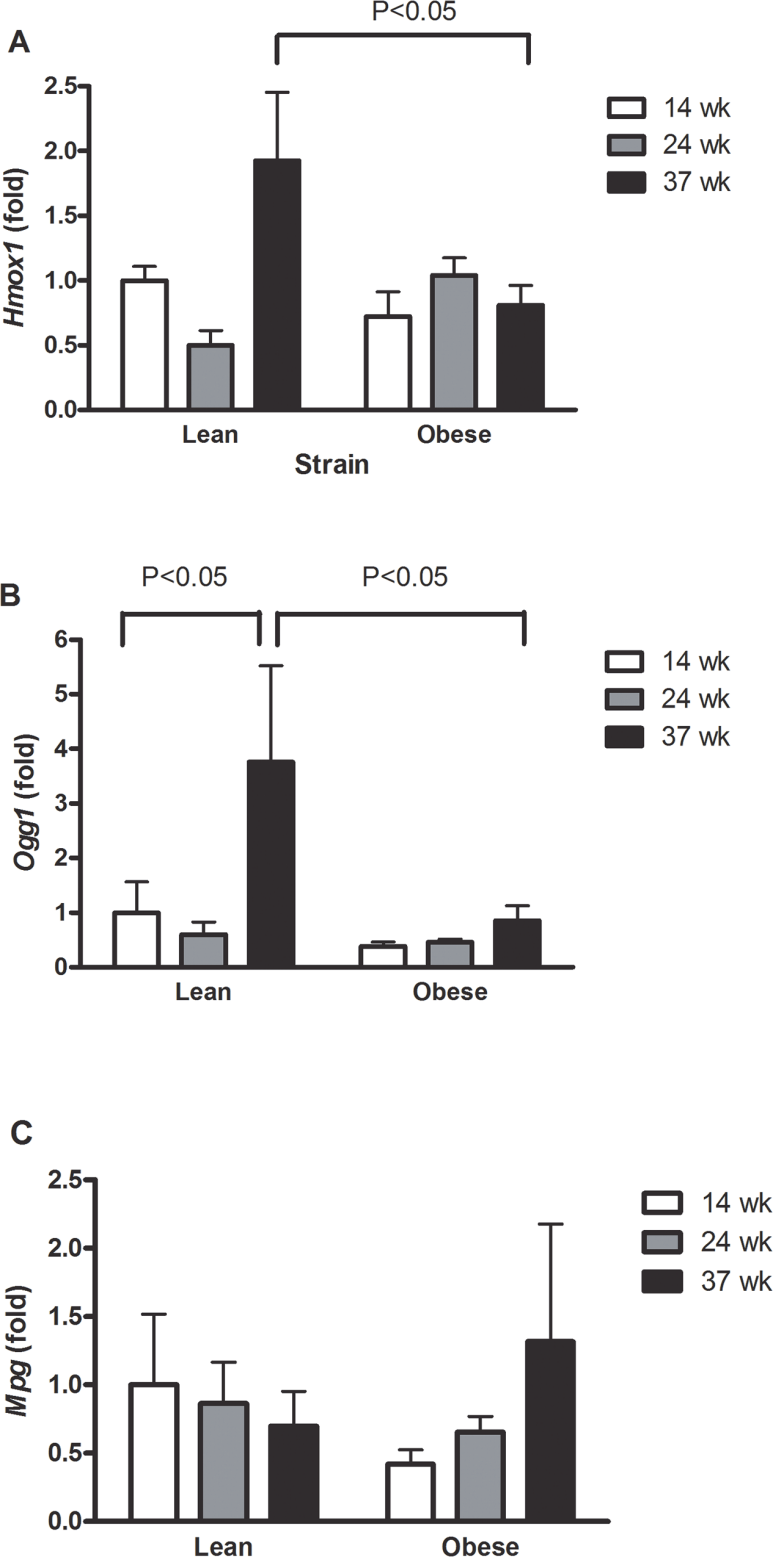
Expression of *Hmox1* (A), *Ogg1* (B) and *Mpg* (C) in the liver of lean and obese Zucker rats. The results are shown as mean and SEM (n = 6–8 per group).

There was increased gene expression of *Hmox1* in the liver of lean rats as compared to the obese counterparts (Fig. 2A, P<0.05). The expression of *Ogg1* in lean rats at 37 weeks of age was increased as compared to both 14 weeks lean rats and 37 weeks obese rats (Fig. 2B, P<0.05, single-factor effect of age). The gene expression of *Mpg* was unaltered (Fig. 2C, P>0.05).

### Both lean and obese Zucker rats have accumulation of HMOX-1 in the liver at week 37 as compared to week 10

**Fig 3:**
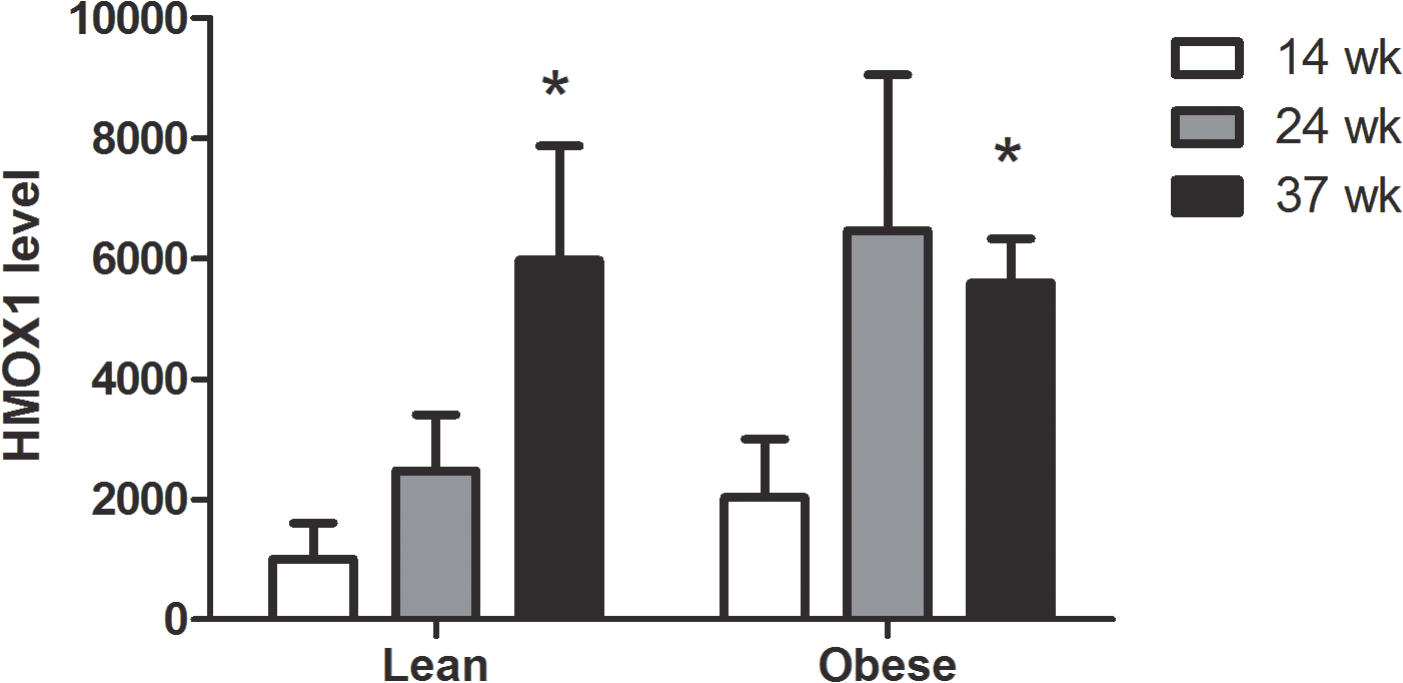
HMOX-1 protein the liver of lean and obese lean Zucker rats. The results are represented as mean ± SEM (n = 5 per group) relative band intensity in Western blot. *Statistical significantly increased compared to young Zucker rats.

Fig. 3 depicts the protein levels of HMOX-1 in liver tissue. For both strains of rats there were higher protein levels of HMOX-1 in the liver at week 37 as compared to week 10 (P<0.05), whereas there were no difference in the expression level between the strains (P = 0.21).

### Lean rats have increased expression of *Srebp2*, whereas obese Zucker rats have substantially decreased expression of cholesterol transporter proteins *Abcg5* and *Acbg8*

**Fig 4:**
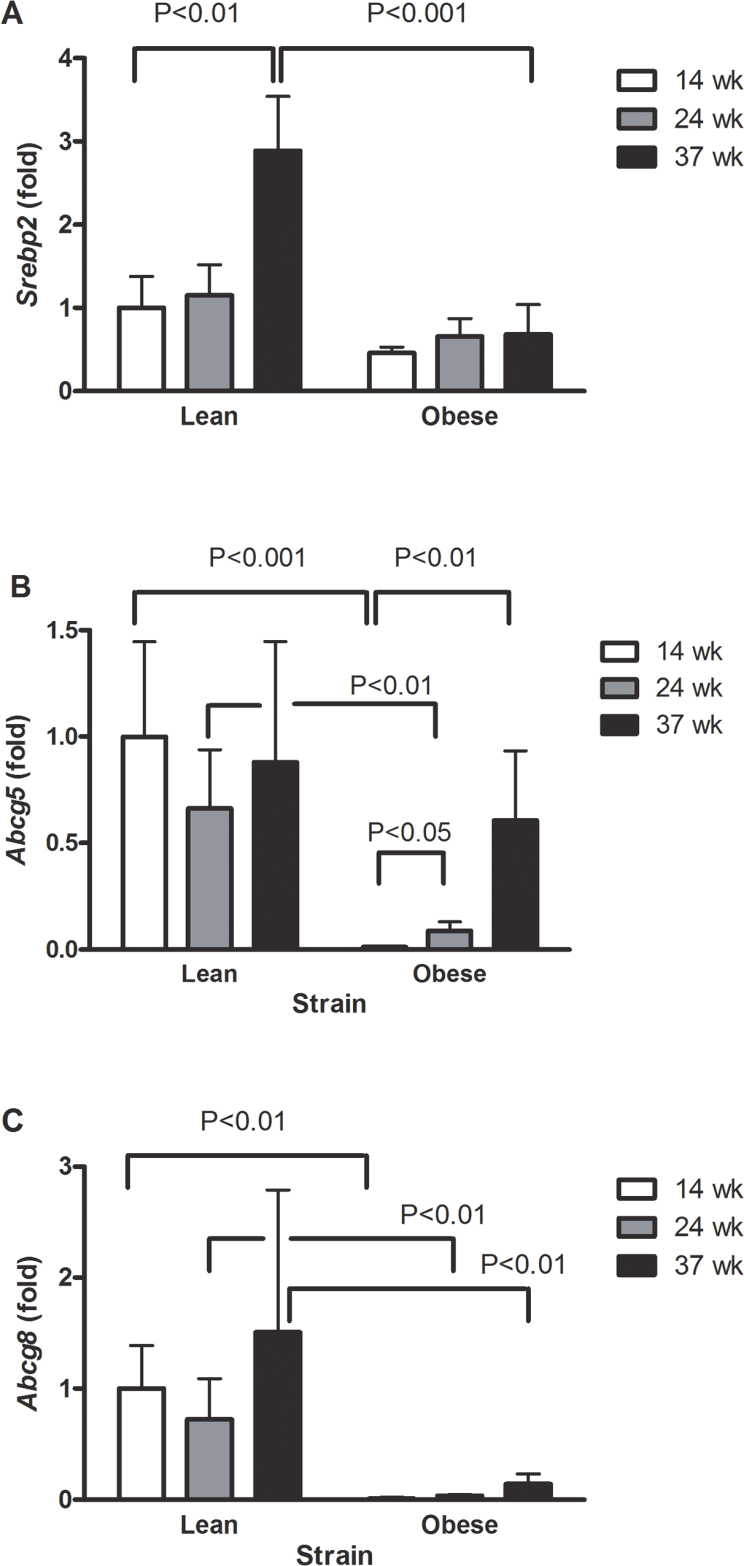
Expression of *Srebp2* (A), *Abcg5* (B) and *Abcg8* (C) in the liver of lean and obese Zucker rats. The results are shown as mean and SEM (n = 5–8 per group).

The expression level of *Srebp2* was increased in 37 weeks old lean rats as compared to the 14 weeks lean rats (P<0.01 and 37 weeks obese rats (P<0.001) (Fig. 4A).There was a decreased gene expression of *Abcg5* in the obese animals (Fig. 4B, P<0.001). The expression level of *Abcg8* was higher at 14 weeks (P<0.001) and 24 weeks (P<0.05) in the lean rats as compared to obese rats at the same age, respectively (Fig. 4C).

### Obese Zucker rats display outward remodelling in the mesenteric arteries

**Fig 5:**
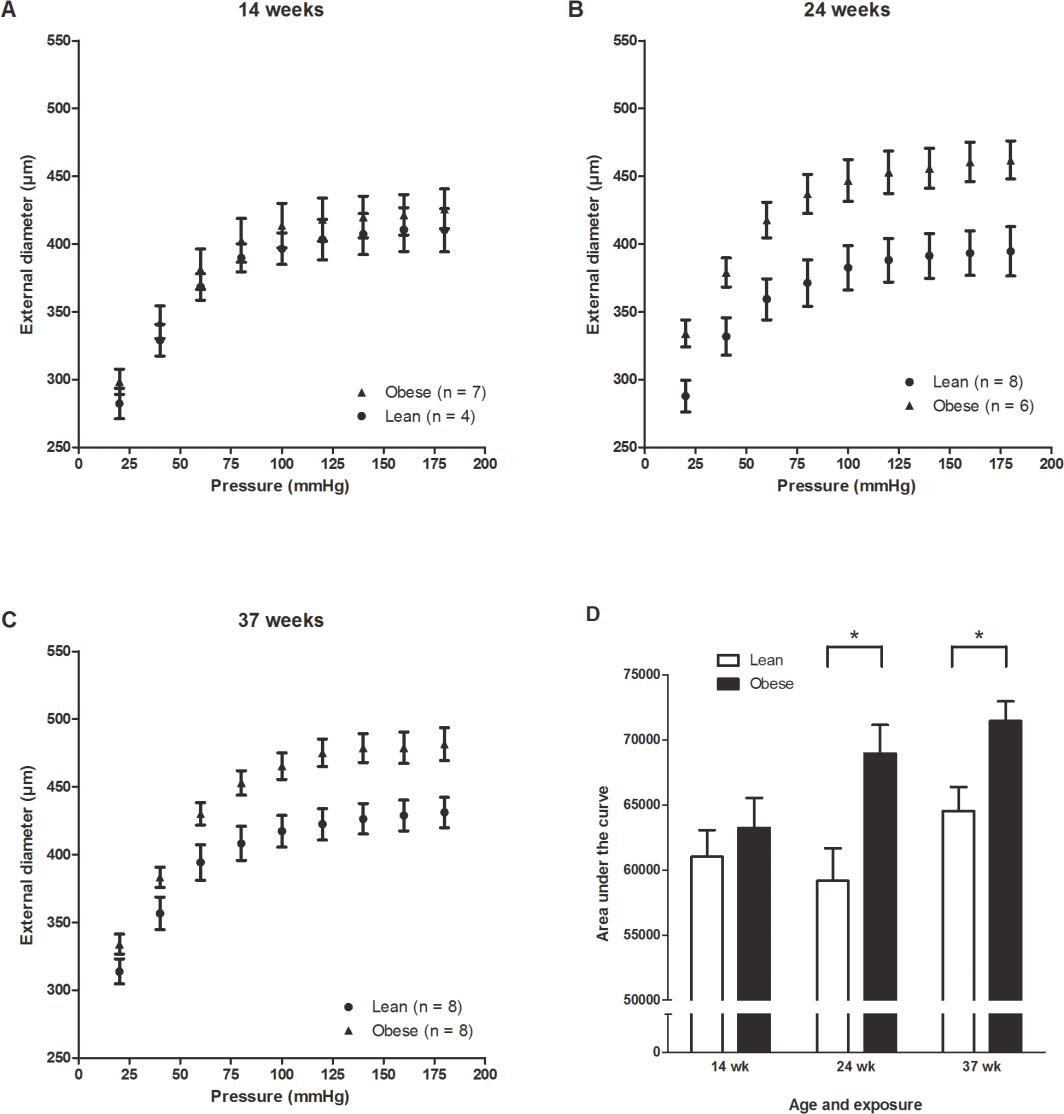
Pressure-diameter relationship measured by increasing the intraluminal pressure from 20 mmHg increments from 20 mmHg to 180 mmHg in rats at 14 (A), 24 (B) or 37 weeks (C) of age. The AUC values are shown in (D). *Statistical significantly increased compared to young obese Zucker rats (mean and SEM).

Fig. 5 shows the pressure-diameter relationship in the lean and obese Zucker rats. The increase in the intraluminal pressure was associated with an increase in the external vessel diameter of the isolated mesenteric arteries, indicating that a myogenic response was not activated under these circumstances. The external diameter of the pressurized mesenteric arteries from lean Zucker rats did not increase significantly with age, whereas the obese Zucker rats at the age of 24 (P<0.05) and 37 weeks (P<0.01) had larger external diameter than the younger rats (Fig. 5A-C). In addition, the obese Zucker rats had a larger external diameter than the lean counterparts at 24 (P<0.01) and 37 (P<0.05) weeks of age (Fig. 5D).

**Fig 6:**
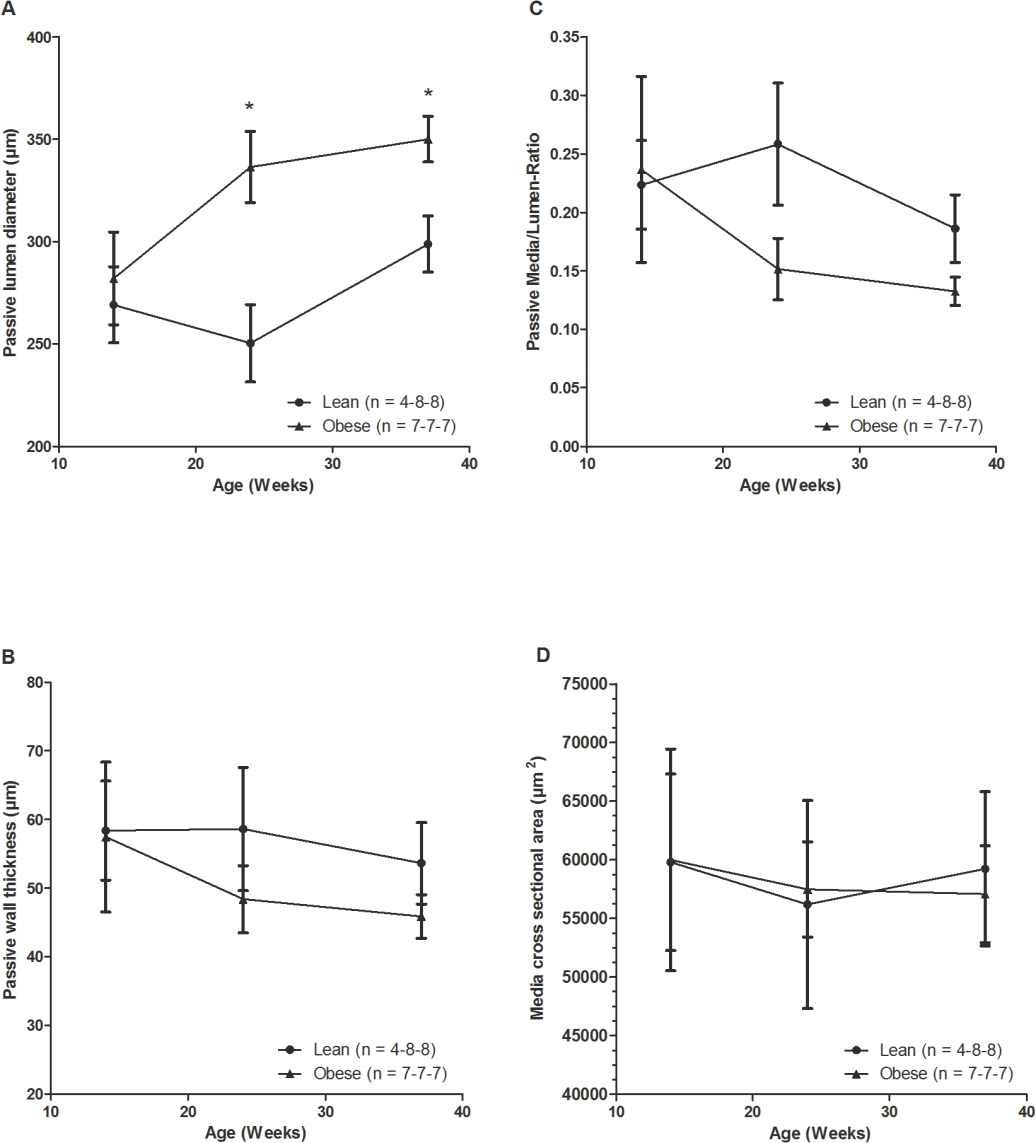
Vessel lumen diameter (A), wall thickness (B), media/lumen ratio (C), and media cross sectional area (D) at 60 mmHg in Ca^2+^-free medium. *Statistically significant increase compared to lean counterparts (mean and SEM).

To investigate the passive structural properties the external and lumen diameters were measured in the presence of Ca^2+^ free extracellular medium, and the wall thickness (WT), media/lumen-ratio (M/L) and media cross sectional area (CSA) were estimated at a distending pressure of 60 mm Hg in all groups. The lumen diameter increased significantly with age in the obese Zucker rats (Fig. 6A), whereas there were no significant changes in the WT (Fig. 6B), M/L (Fig. 6C), and CSA (Fig. 6D). These results indicate the presence of outward eutrophic remodelling of the vascular wall in the obese Zucker rats at weeks 24 and 37.

**Fig 7:**
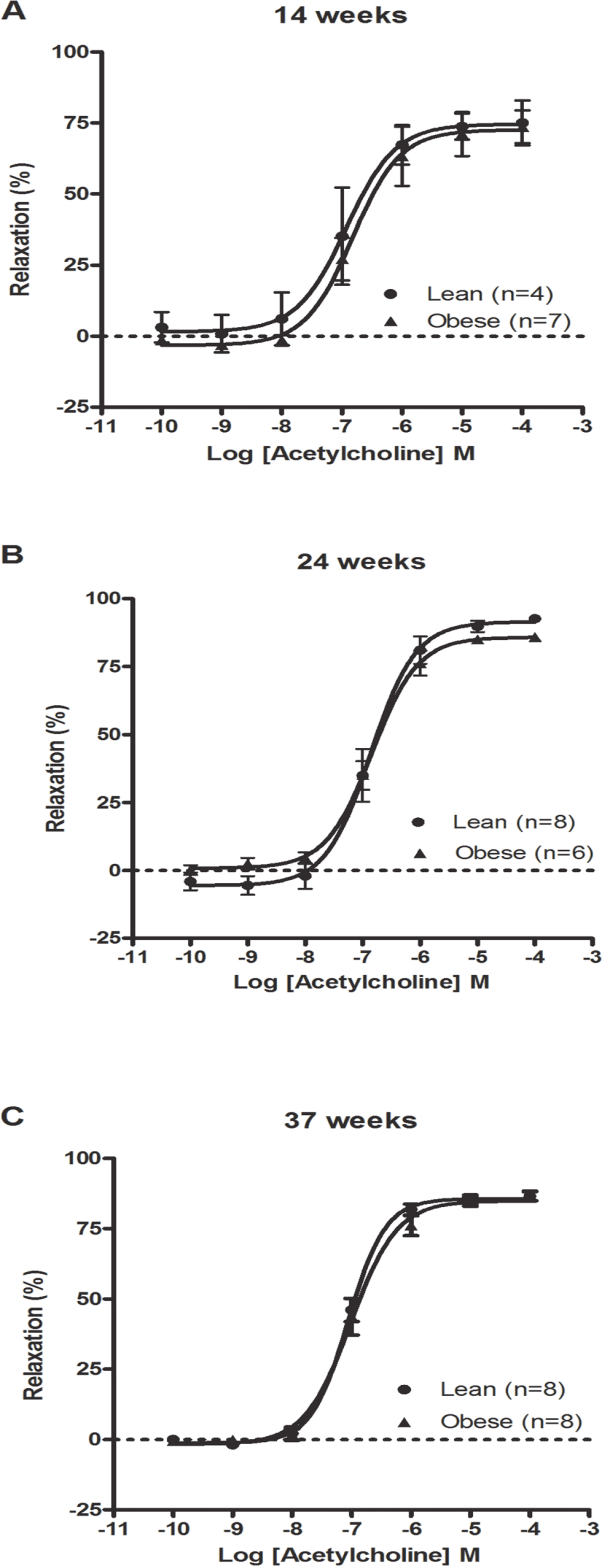
Acetylcholine-mediated endothelium-dependent vasodilatation in pressurized (60 mmHg) mesenteric arteries of rats at 14 (A), 24 (B) or 37 weeks (C) of age. *Statistically significant effect of age and group (pEC_50_ single-factor effect). The results are shown as mean and SEM.

Exposure to acetylcholine induced a concentration-dependent dilatation in mesenteric arteries pressurized at 60 mmHg (Fig. 7A-C). The corresponding pEC_50_ and E_max_ values are reported in Table 2. There were higher E_max_ values at 24 and 37 weeks of age as compared to the corresponding 14 week animals in both lean and obese groups (P<0.05), whereas there was no difference between the two strains. By contrast, the pEC_50_ values were unaltered with regards to both strain and age differences.

**Table 2:** Acetylcholine-mediated endothelium-dependent vasodilatation in pressurised (60 mmHg) second-order mesenteric arteries of rats at 14, 24 or 37 weeks of age.

Age	pEC_50_		E_max_ (%of maximal relaxation)	
	Lean	Obese	Lean	Obese
14 weeks	-7.03 ± 0.36 (4)	-6.71 ± 0.23 (7)	72.9 ± 5.2 (4)	73.4 ± 5.6 (7)
24 weeks	-6.85 ± 0.15 (8)	-6.80 ± 0.13 (6)	91.2 ± 1.3 (8)*	86.0 ± 0.9 (6) *
37 Weeks	-7.04 ± 0.14 (8)	-6.7 ± 0.10 (8)	85.5 ± 1.8 (8) *	85.4 ± 1.8 (8) *

Mean ± SEM (n).

*P<0.05 compared with the corresponding 14 weeks old rats.

## Discussion

The results of the present study showed increased levels of DNA strand breaks in the liver of lean Zucker rats, whereas the same group had an age-dependent decrease in levels of oxidatively damaged DNA that occurred concurrently with increased gene expression of *Ogg1* and *Hmox1* and HMOX-1 protein levels in the liver. By contrast, mesenteric arteries from the obese rats exhibited outward remodelling without vessel wall hypertrophy, with similar responsiveness to acetylcholine-mediated vasodilatation as lean counterparts.

The obese rats in the present study displayed altered lipid metabolism, including high serum concentrations of triglycerides and cholesterol as well as increased hepatic lipid load. The unaltered serum levels of ALT indicate that metabolic alteration occurred without hepatic cytotoxicity. There was also indication of intrinsic hepatic dysregulation of sterol transport in terms of down regulation of *Abcg5* and *Abcg8*. The unaltered non-fasting glucose concentrations and the concurrent increase in insulin concentrations in the serum of the obese rats suggest t insulin resistance.

It is well-known that cells respond to oxidative stress by increasing the expression of *Hmox1*, although the reason for increasing catabolism of heme is unclear as a cellular protection mechanism [9]. The observations of increased HMOX-1 protein expression and decreased levels of oxidatively damaged DNA in the liver of lean rats suggest a cellular protection. This is supported by earlier observations in which 12 week old Zucker rats had increased hepatic expression of *Hmox1* and enzyme activity following inhalation of isoflurane, whereas no difference in the hepatic *Hmox1* expression between lean and obese rats was noted [24]. *Hmox1* is regulated by nuclear factor erythroid-2 related factor-2 (Nrf2), although Nrf2-independent pathways of *Hmox-1* regulation do exist [9]. Nrf2 is involved in the regulation of genes in oxidant-defence and redox signalling [25]. It has been shown that increased Nrf2 activity is associated with a higher level of hepatic steatosis in leptin-deficient obese mice [26]. Increased *Nrf2* expression was also observed in young (3 months) LDL receptor knockout mice on a high-fat diet, whereas middle aged mice (12 months) with a more pronounced hepatic steatosis had unaltered expression of *Nrf2* [27]. However, mice fed a high-fat diet had decreased hepatic lipid accumulation following exposure to a synthetic oleanolic triperpenoid compound with strong Nrf2 inducing activity [28]. It has also been shown that rats fed on a rich fat and carbohydrate diet (fructose and sucrose) for 8 weeks displayed hepatic lipid accumulation and injury (increased plasma levels of ALT and AST), where the expression levels of *Nrf2* and *Hmox1* were decreased [29]. Additionally, mice which were genetically altered to express either low or high Nrf2 activity did not differ in the extent of hepatic triglyceride content when fed on a high-fat diet [30]. Nrf2 deficient mice on a high-fat diet had the same extent of hepatic lipid accumulation as wild-type animals, whereas they displayed increased inflammation and tissue injury in terms of plasma ALT and AST [31]. Another study also investigated the effect of high-fat diet in Nrf2 deficient mice and showed increased hepatic lipid accumulation, inflammation and tissue injury in terms of plasma ALT and AST in comparison to wild-type mice [32].

Here, the level of DNA damage was assessed as a biomarker of oxidative stress. We have previously observed an age-dependent increase in the level of DNA strand breaks in the liver of dyslipidemic apolipoprotein E knockout mice, whereas wild type mice had unaltered levels of DNA strand breaks [33,34]. There is compelling evidence for age-dependent accumulation of oxidatively damaged DNA in wild type animal organs [35]. However, the age span in the present study was relatively narrow in comparison to previous studies on wild type mice that have typically used a gap of 1 year or more in difference between the youngest and oldest group of animals. An earlier study showed that obese Zucker rats had higher levels of liver DNA strand breaks in comparison to lean Zucker rats at 3, 6 and 15 months [36]. It is possible that the modest age-dependent increase in level of DNA strand breaks in the liver of the lean rats was due to the narrow age span in the present study. The expression level of *Hmox1* and HMOX-1 protein also increased age-dependently in the lean rats, which suggests that the increased level of DNA strand breaks was not a coincidence. It should be noted that our observations are not in contradiction to earlier studies in which signs of oxidative stress in the liver of obese Zucker rats and hepatic cytotoxicity (increased serum ALT levels) were observed. For instance, it was shown that 23 weeks old obese Zucker rats had lower levels of glutathione as well as reduced activity of catalase, glutathione peroxidase and superoxide dismutase in the liver, although this was also associated with increased serum levels of ALT [37,38].

It was somewhat surprising that the level of oxidatively damaged DNA decreased with age in the lean rats and that the young obese Zucker rats had lower levels of oxidatively damaged DNA than the lean counterparts. In the lean rats this can be explained by increased expression level of *Ogg1*. We have previously observed inverse relationships between levels of oxidatively damaged DNA and expression of *Ogg1* in the lung and liver of rats exposed to particulate matter and ionizing radiation [39–43]. Here, the increased expression of *Ogg1* seems to be a differential effect related to the repair of small oxidized DNA lesions such as 8-oxodG, highlighted by the fact that no alteration in the expression of *Mpg* which is involved in the response to other types of DNA lesions. Other studies have shown that 22 weeks old lean and obese Zucker rats had the same level of immunostaining of 8-oxodG in the liver, whereas lean rats on a choline-deficient diet had higher 8-oxodG immunostaining than obese Zucker rats [44]. This is further supported by observations of the same level of 8-oxodG immunostaining in the kidney and pancreas of 26 weeks lean or obese Zucker rats [45]. However, it should be noted that these antibody-based detection methods of 8-oxodG are regarded as being highly unspecific as evidenced by measurements of the background level of 8-oxodG that are orders of magnitude higher than the widely accepted levels of the lesion based on chromatographic and/or enzymic detection [46]. Collectively, our data suggest that hepatic steatosis as seen in obese Zucker rats is not associated with oxidative stress to DNA. Nevertheless, decreased levels of oxidatively damaged DNA occurred concurrently with increased expression of *Ogg1* and seemingly unaltered steady-state levels of base lesions, possibly at the expense of increased levels of DNA strand breaks.

The age-dependent increase in external and passive lumen diameter in the obese rats indicates outward remodelling of the mesenteric arteries. This occurred irrespective of acetylcholine-mediated endothelium-dependent vasodilatation, indicating that outward remodelling in the obese Zucker rats was not caused by endothelial dysfunction. It has been reported previously that mesenteric arteries of 20 week old obese Zucker rats had the same acetylcholine-mediated vasodilatation response as lean Zucker rats and the differences in E_max_ and pEC_50_ of acetylcholine concentration-response curves between lean and obese rats at 32 weeks of age were not statistically significant [47]. However, an age-dependent progression of outward remodelling in mesenteric arteries also has been observed in wild type rats at the age 2 to 26 months [48]. Furthermore, it has been demonstrated that mesenteric arteries from 15–18 week old obese Zucker rats had blunted endothelial-dependent vasomotor dysfunction without remodelling [49], and third order mesenteric arteries from 25 weeks old obese Zucker rats had impaired acetylcholine-mediated vasodilatation [50]. Moreover, outward remodelling and decreased responsiveness to acetylcholine-mediated vasodilatation in first order mesenteric arteries was observed with high-flow condition by ligation of adjacent lateral vessels in obese Zucker rats [51]. The acetylcholine-mediated vasodilatation was attenuated in pressurised coronary arteries and to a lesser extent in aorta segments and pressurised mesenteric arteries with advancing age and/or manifestation of diabetes in Zucker rats [52]. In this context, our experimental system differs somewhat from earlier studies with regards to the type of vessel, low-diabetic condition and age of the rats. In this study, it is plausible that a larger diameter of the mesenteric second order arteries increasing with age is related to generally higher blood flow required to sustain the growth of the obese Zucker rats. The development of more severe steatosis induced by a methionine-choline deficient diet has also been associated with increased portal pressure and endothelial dysfunction in the liver [4], whereas severe liver cirrhosis was associated with endothelial dysfunction in the mesenteric arteries [5]. The vascular effect in the present study should be viewed in similar light with our previous data where we observed decreased acetylcholine- and nitroglycerin-mediated vasorelaxation in aorta segments (isometric preparations using wire myograph setup) from the obese Zucker rats at the age of 24 and 37 weeks, whereas there was no difference at the age of 14 weeks [17]. These observations are in agreement with a study of 32 weeks old Zucker rats, which had impaired acetylcholine-mediated vasorelaxation in aorta segments, whereas no difference in acetylcholine-mediated vasorelaxation of mesenteric arteries from the same rats was noted [53]. All these studies have used variably sized vessels and animals ranging in age, diet and experimental conditions, which might explain some of the differences reported in terms of vascular functions between obese and lean Zucker rats. However, our results are consistent with the perception that the vasomotor function of mesenteric arteries appear less sensitive than the aorta and coronary arteries to metabolic syndrome changes evolving in obese Zucker rats.

In conclusion, this study showed that obese Zucker rats had increased hepatic lipid load without a clear indication of oxidative stress-generated DNA damage. There was outward remodelling of second order mesenteric arteries that was not associated with acetylcholine-controlled endothelium-dependent vasomotor dysfunction.
